# Hog1 Regulates Stress Tolerance and Virulence in the Emerging Fungal Pathogen Candida auris

**DOI:** 10.1128/mSphere.00506-18

**Published:** 2018-10-24

**Authors:** Alison M. Day, Megan M. McNiff, Alessandra da Silva Dantas, Neil A. R. Gow, Janet Quinn

**Affiliations:** a Institute for Cell and Molecular Biosciences, Faculty of Medicine, Newcastle University, Newcastle upon Tyne, United Kingdom; b MRC Centre for Medical Mycology, Institute of Medical Sciences, University of Aberdeen, Aberdeen, United Kingdom; Carnegie Mellon University

**Keywords:** *Candida auris*, pathogenesis, stress adaptation, stress kinases

## Abstract

Candida auris has recently emerged as an important, multidrug-resistant fungal pathogen of humans. Comparative studies indicate that despite high levels of genetic divergence, C. auris is as virulent as the most pathogenic member of the genus, Candida albicans. However, key virulence attributes of C. albicans, such as morphogenetic switching, are not utilized by C. auris, indicating that this emerging pathogen employs alternative strategies to infect and colonize the host. An important trait required for the pathogenicity of many fungal pathogens is the ability to adapt to host-imposed stresses encountered during infection. Here, we investigated the relative resistance of C. auris and other pathogenic *Candida* species to physiologically relevant stresses and explored the role of the evolutionarily conserved Hog1 stress-activated protein kinase (SAPK) in promoting stress resistance and virulence. In comparison to C. albicans, C. auris is relatively resistant to hydrogen peroxide, cationic stress, and cell-wall-damaging agents. However, in contrast to other *Candida* species examined, C. auris was unable to grow in an anaerobic environment and was acutely sensitive to organic oxidative-stress-inducing agents. An analysis of C. auris *hog1*Δ cells revealed multiple roles for this SAPK in stress resistance, cell morphology, aggregation, and virulence. These data demonstrate that C. auris has a unique stress resistance profile compared to those of other pathogenic *Candida* species and that the Hog1 SAPK has pleiotropic roles that promote the virulence of this emerging pathogen.

**IMPORTANCE** The rapid global emergence and resistance of Candida auris to current antifungal drugs highlight the importance of understanding the virulence traits exploited by this human fungal pathogen to cause disease. Here, we characterize the stress resistance profile of C. auris and the role of the Hog1 stress-activated protein kinase (SAPK) in stress resistance and virulence. Our findings that C. auris is acutely sensitive to certain stresses may facilitate control measures to prevent persistent colonization in hospital settings. Furthermore, our observation that the Hog1 SAPK promotes C. auris virulence akin to that reported for many other pathogenic fungi indicates that antifungals targeting Hog1 signaling would be broad acting and effective, even on emerging drug-resistant pathogens.

## Abstract (precis)

The rapid global emergence and resistance of Candida auris to current antifungal drugs highlight the importance of understanding the virulence traits exploited by this human fungal pathogen to cause disease. Here, we characterize the stress resistance profile of C. auris and the role of the Hog1 stress-activated protein kinase (SAPK) in stress resistance and virulence. Our findings that C. auris is acutely sensitive to certain stresses may facilitate control measures to prevent persistent colonization in hospital settings. Furthermore, our observation that the Hog1 SAPK promotes C. auris virulence akin to that reported for many other pathogenic fungi indicates that antifungals targeting Hog1 signaling would be broad acting and effective, even on emerging drug-resistant pathogens.

## INTRODUCTION

The emerging fungal pathogen Candida auris was first reported in Japan in 2009 (1) and, in less than a decade, has been isolated from patients in multiple countries spanning five continents (reviewed in reference 2). A number of attributes of this fungal pathogen cause concern, such as widespread multidrug resistance, transmission within hospital settings, and an association with high mortality rates. Such high mortality rates are likely related to the observations that C. auris infections are largely hospital acquired and mainly affect critical care patients, whereas the ability of C. auris to trigger hospital outbreaks is likely related to the persistent colonization of both hospital wards and patients with this fungus (3, 4). The majority of clinical C. auris isolates are resistant to fluconazole, the most widely prescribed prophylactic antifungal treatment. Disturbingly, a number of C. auris strains have been isolated that are resistant to all three classes of antifungal drugs currently available for the treatment of systemic infections, thereby severely limiting treatment options (5). This potential problem in treating C. auris infections underscores the importance of rapid infection prevention and the implementation of control measures to curb such outbreaks and highlights the need to investigate the pathobiology of this emerging pathogen.

Genomic analyses revealed that C. auris is phylogenetically related to Candida lusitaniae and Candida haemulonii but is highly diverged from major pathogenic species, including Candida albicans and Candida glabrata (6). Interestingly, the sequencing of multiple isolates revealed C. auris to be separated into 4 distinct geographic clades, namely, the South Asian, East Asian, South African, and South American clades, which are separated by tens of thousands of single polynucleotide polymorphism differences (5). Within each clade, however, there are minimal genetic differences (5, 7), indicating that C. auris independently emerged in different geographic locations at around the same time. The trigger responsible for such simultaneous emergence is unclear, but the increasing use of prophylactic antifungal agents, to which C. auris is resistant, may be a factor (8). The C. auris genome is between 12.1 and 12.7 Mb (5–7, 9), with approximately 5,500 protein-encoding genes (9). An initial study indicated that the C. auris genome was diploid (6); however, recent Illumina sequencing of the C. auris genome has provided strong evidence that C. auris is haploid (9). Indeed, the haploid nature of C. auris was confirmed in a recent study in which a single disruption event was sufficient to delete the catalase-encoding gene, with consequential peroxide sensitivity (10).

To gain insight into the pathobiology and virulence of C. auris, comparative studies with the most pathogenic *Candida* species, Candida albicans, have been performed. In both an invertebrate Galleria mellonella infection model (11) and a murine model of systemic candidiasis (12), C. auris displayed a similar level of virulence as C. albicans. Subsequently, investigations have been undertaken to determine whether C. auris employs the same battery of virulence traits as C. albicans, including morphogenetic switching, adhesion, the production of secreted enzymes, and biofilm formation (13). While no evidence of morphogenetic switching was observed, a number of the isolates tested did secrete phospholipase and protease enzymes, albeit at generally lower levels than for C. albicans (13). Moreover, C. auris was much less adherent than C. albicans to solid surfaces (13), which may be related to the significantly fewer adhesin-encoding genes in the C. auris genome (6). Similarly, although C. auris formed biofilms, these were much less dense than those formed by C. albicans (13, 14). Collectively, these observations indicate that C. auris may utilize different strategies to promote virulence than those exploited by the phylogenetically divergent pathogen C. albicans. Intriguingly, a recent study revealed that C. auris is not effectively recognized by neutrophils and thus evades neutrophil-mediated killing, which in turn may contribute to the ability of this fungus to cause disease (15).

An additional trait that is required for the virulence of diverse pathogenic fungi is the ability to respond and adapt to the changing microenvironments within the host (16). Niches colonized within the human host are dynamic, in that they display fluctuations in osmolarity, pH, reactive oxygen and nitrogen species, and the availability of macro- and micronutrients (16). In addition, in certain niches such as in the gut, an anaerobic environment is encountered. Central to stress sensing and signaling in pathogenic fungi is the Hog1-related stress-activated protein kinase (SAPK), which was originally identified in the model yeast Saccharomyces cerevisiae as being essential for osmoadaption (17). Such SAPKs are essential for fungal survival against host-imposed stresses and are key virulence determinants in human-, plant-, and insect-infecting fungal pathogens (18–20). However, with the exception of a recent study (21) revealing that C. auris is relatively resistant to reactive oxygen species, little else is known regarding the ability of this pathogen to sense and respond to physiologically relevant stresses. Here, we compared the stress resistance profile of C. auris with those of other *Candida* species and explored the role of the conserved Hog1 SAPK in stress signaling and virulence in this important emerging pathogen.

## RESULTS

### Stress resistance phenotypes of C. auris.

An increased resistance to the triazole fluconazole (MIC > 64 mg/liter) has been reported in a high proportion of C. auris isolates across all 4 geographical clades (2, 5). However, little is known regarding the ability of C. auris to resist physiological stresses encountered in the host. Here, we investigated the ability of C. auris to grow in the presence of physiologically relevant stresses, including reactive oxygen species (ROS), cationic stress, acid and alkaline stresses, and cell-wall-damaging agents. We also investigated whether C. auris is able to grow in an anaerobic environment. Three C. auris isolates were examined: NCPF8985 (C. auris-1), a multidrug-resistant isolate from the Indian clade; NCPF8971 (C. auris-2), a nonaggregating strain from the Indian clade; and NCPF8977 (C. auris-3), an aggregating strain from the South African clade. The relative stress resistances of these C. auris isolates were compared against those exhibited by C. albicans, which is the most clinically important *Candida* species (22), Candida dubliniensis, which is a generally less stress-tolerant close relative of C. albicans (23), and C. glabrata, which is phylogenetically divergent from C. albicans and, similar to C. auris, highly drug resistant (24).

**FIG 1:**
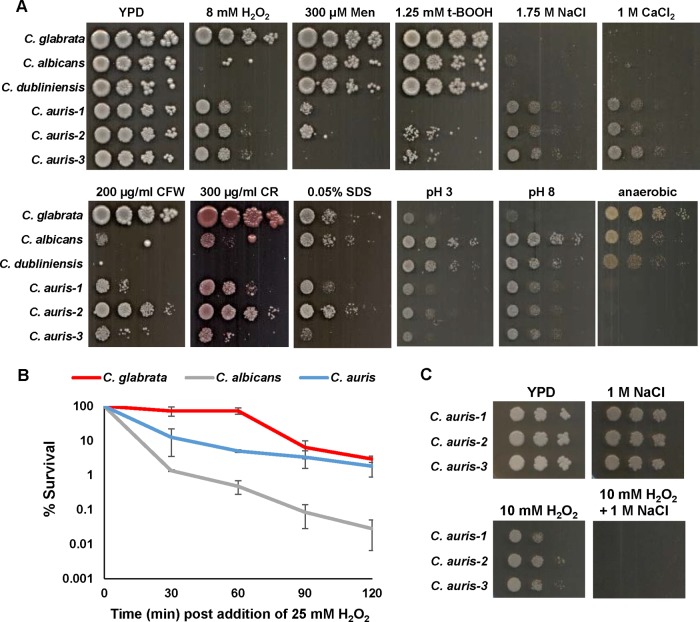
Stress resistance comparisons. (A) C. auris has a unique stress resistance profile. Exponentially growing *Candida* strains were spotted in serial dilutions onto YPD agar plates containing the indicated additives and incubated for 24 or 48 h at 30°C. (B) C. auris is more resistant than C. albicans to H_2_O_2_. Exponentially growing *Candida* strains were treated with 25 mM H_2_O_2_, and cell survival at the indicated times was calculated as described in Materials and Methods. (C) C. auris is sensitive to combinatorial H_2_O_2_ and cationic stress. Exponentially growing strains and 10-fold dilutions thereof were spotted onto the indicated plates and incubated at 30°C for 24 h.

On solid medium (Fig. 1A) and in liquid culture (Fig. 1B), C. auris was clearly more resistant to oxidative stress imposed by H_2_O_2_ than C. albicans and C. dubliniensis. However, C. glabrata displayed the greatest tolerance to H_2_O_2_, and such high levels of resistance have previously been noted (25). In contrast, C. auris was much less tolerant than all other species tested to the superoxide-generating agent menadione and the organic peroxide *tert*-butyl hydroperoxide (Fig. 1A) and yet displayed the highest levels of resistance to cationic stress imposed by either sodium chloride or calcium chloride. As previous studies have shown that a range of fungi, including C. albicans and C. glabrata, are acutely sensitive to combinations of H_2_O_2_ and cationic stresses (26, 27), we examined whether C. auris was also sensitive to such combinatorial stress treatments. As shown in Fig. 1C, C. auris isolates grew in the presence of either 10 mM H_2_O_2_ or 1 M NaCl. However, the growth was abolished upon simultaneous exposure to both stresses, illustrating that C. auris is also sensitive to combinatorial oxidative and cationic stress treatments.

Upon growth in the presence of cell-wall-damaging agents, C. auris was more resistant to the chitin-binding dye calcofluor white and the β-1,3-glucan-binding dye Congo red than C. albicans and C. dubliniensis, suggesting that there are possible cell wall differences between these species. However, C. auris was much less able to adapt to acidic or alkaline pH environments than C. albicans and C. dubliniensis, although C. glabrata displayed the greatest impairment of growth. Interestingly, C. auris failed to grow under anaerobic conditions (Fig. 1B), in contrast to the other *Candida* species tested (Fig. 1B). Collectively, this phenotypic analysis illustrates that C. auris has a unique stress resistance profile compared to those of the three pathogenic *Candida* species examined here.

### Identification and functional characterization of C. auris Hog1.

**FIG 2:**
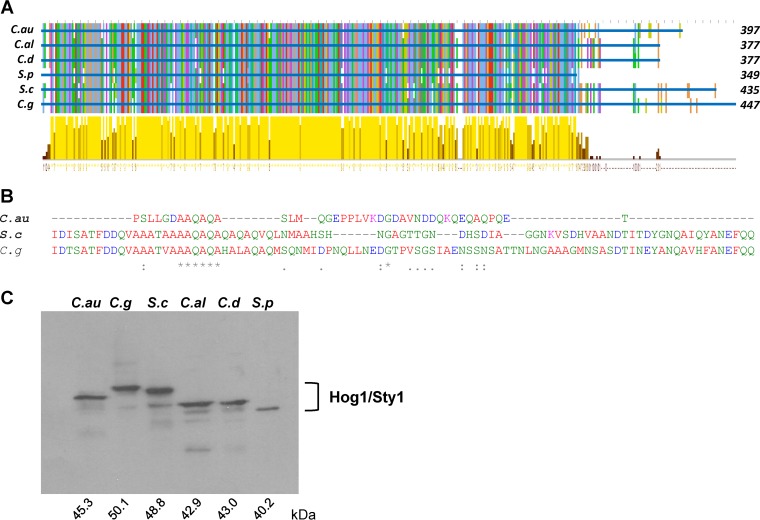
Sequence comparisons of fungal HOG1 orthologues. (A) Multiple-sequence alignment of the indicated Hog1 sequences using Clustal Omega and visualized using Jalview. The top graph shows the sequence conservation: columns are highlighted with the same color where there is conservation across the compared sequences. The graph at the bottom tracks the conservation with identical amino acid sequences highlighted in yellow. *C. au*; C. auris, *C. al*; C. albicans, *C. d*; C. dubliniensis; *S. p*; S. pombe, *S. c*; S. cerevisiae, *C. g*; C. glabrata. (B) Sequence alignment of the C-terminal Hog1 sequences from C. auris, S. cerevisiae, and C. glabrata. (C) Hog1 mobility. Western blot depicting the size of Hog1 orthologues from the indicated fungal species. The predicted size of each orthologue is shown (kDa).

The Hog1 SAPK is among the most conserved stress-sensing and signaling proteins across diverse fungal species (28) and is a key virulence factor in many human fungal pathogens (18, 29–31). A BLASTP search of the draft C. auris genome sequence (6) identified an open reading frame that was 87% identical to the C. albicans Hog1 sequence. A multiple alignment of Hog1 sequences from C. auris, C. albicans, C. dubliniensis, and C. glabrata, in addition to those from the model yeasts Saccharomyces cerevisiae and Schizosaccharomyces pombe, was performed using Clustal Omega (32). The condensed alignment is shown in Fig. 2A and the full alignment is in Fig. S1 in the supplemental material. There is a very high level of homology throughout the Hog1 kinase domain, which spans the first 300 amino acids (Fig. 2A). In addition, the common docking (CD) domain (residues 302 to 316) and the Pbs2 binding domain (residues 320 to 350) characterized in S. cerevisiae Hog1 (33) and located immediately downstream of the kinase domain are conserved in all Hog1 orthologues examined here (Fig. S1). Notably, the two critical aspartic acid amino acids within the S. cerevisiae CD, which mediate interactions with the Pbs2 activating kinase, the Ptp2 inactivating phosphatase, and the Rck2 substrate, are conserved within all fungal SAPK orthologues, including C. auris Hog1 (Fig. S1). The only divergent region within these fungal SAPK orthologues is at the C terminus. This C-terminal extension is largest in C. glabrata (95 residues) and S. cerevisiae (83 residues), of intermediate length in C. auris (42 residues) and in C. albicans and C. dubliniensis (22 residues), and absent in S. pombe. In S. cerevisiae, this region may function to prevent autoactivation of the kinase (34). Whether such a function is conserved in C. auris Hog1 is unclear due the limited homology with the C-terminal regions (Fig. 2B). Nonetheless, the variation in the lengths of the C-terminal regions correlates with size differences between the fungal SAPKs as detected by Western blotting (Fig. 2C).

10.1128/mSphere.00506-18.1 FIG S1 Sequence alignment of fungal *HOG1* orthologs. Multiple-sequence alignment of the indicated Hog1 sequences using Clustal Omega. The conserved TGY phosphorylation motif is shown in bold. The CD domain is highlighted in yellow with the two conserved aspartic acid residues in bold, and the Pbs2-binding domain is highlighted in blue. *C. au*; C. auris, *C. al*; C. albicans, *C. d*; C. dubliniensis; *S. p*; S. pombe, *S. c*; S. cerevisiae, *C. g*; C. glabrata. Download FIG S1, TIF file, 1.9 MB. Copyright © 2018 Day et al. 2018 Day et al. This content is distributed under the terms of the Creative Commons Attribution 4.0 International license.

**FIG 3:**
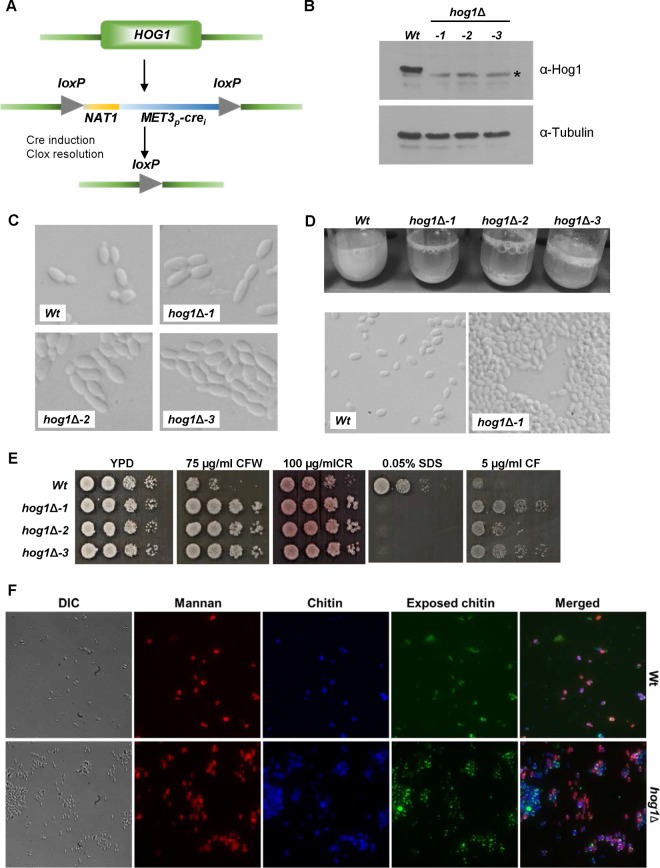
Construction and analysis of *C. auris hog1*Δ cells. (A) Schematic diagram of the strategy used to delete *HOG1*. (B) Western blotting of potential *hog1*Δ strains, identified by PCR genotyping, confirmed the deletion of Hog1. Western blot analysis of lysates prepared from the indicated strains and probed with an anti-Hog1 antibody. *, nonspecific band present in all extracts.(C) Deletion of *HOG1* impacts C. auris cell morphology. DIC images of exponentially growing wild-type and *hog1*Δ C. auris strains. (D) C. auris cells lacking *HOG1* aggregate. Micrographs of wild-type and *hog1*Δ strains grown overnight in YPD medium. Images of culture tubes demonstrate the rapid sedimentation of cells lacking *HOG1*. (E) Deletion of *HOG1* impacts resistance to cell-wall-damaging agents. Exponentially growing strains were spotted onto rich medium plates containing the indicated additives and incubated at 30°C for 24h. (F) C. auris *hog1*Δ cells exhibit more exposed chitin.

To investigate the function of Hog1 in C. auris, deletion strains were constructed in the wild-type background NCPF8985. As detailed in Materials and Methods, this was achieved using the Cre-lox-NAT system (Fig. 3A) developed for use in C. albicans (35). Three independent *hog1*Δ strains were created, and Western blotting confirmed the absence of the Hog1 protein in these mutants (Fig. 3B). Consistent with previous reports (11), wild-type C. auris formed oval yeast cells. However, the deletion of Hog1 resulted in larger elongated cells that clustered together (Fig. 3C). This was particularly evident in overnight cultures, as *hog1*Δ cells sedimented rapidly due to the presence of large aggregates (Fig. 3D). Previously, it was shown that a subset of C. auris clinical isolates form large aggregates upon resuspension in phosphate-buffered saline (PBS), and such aggregates could not be physically disrupted (11). Upon the resuspension of the parental wild-type and *hog1*Δ cells in PBS, only cells lacking Hog1 formed aggregates (see Fig. S2). However, all *hog1*Δ cell aggregates, whether formed during growth in liquid medium or following the resuspension of colonies in PBS, were readily disrupted by sonication, illustrating that the aggregation was due to cell clumping.

10.1128/mSphere.00506-18.2 FIG S2 Aggregation phenotype of *hog1*Δ cells. Micrographs of wild-type and *hog1*Δ cells taken from YPD agar plates and resuspended in PBS. All *hog1*Δ isolates rapidly formed aggregates in contrast to the parental wild-type strain. Download FIG S2, TIF file, 2.9 MB. Copyright © 2018 Day et al. 2018 Day et al. This content is distributed under the terms of the Creative Commons Attribution 4.0 International license.

To explore whether cell wall changes contribute to the aggregation phenotype of *hog1*Δ cells, the sensitivity to cell-wall-damaging agents was examined. As shown in Fig. 3E, *hog1*Δ cells were more resistant to both the β-1,3-glucan binding dye Congo red and the chitin-binding dye calcofluor white than the wild-type cells. Consistent with this, *hog1*Δ cells were also clearly more resistant to the echinocandin antifungal caspofungin, which targets β-1,3-glucan synthase (Fig. 3E). In contrast, the exposure of cells to the anionic detergent SDS, which denatures cell wall proteins and damages lipids, revealed *hog1*Δ mutant cells to be much more sensitive than wild-type cells (Fig. 3E). Collectively, these results indicate significant differences in the cell walls of C. auris wild-type and *hog1*Δ cells. To explore this further, fluorescence microscopy was performed to examine mannan and chitin levels. While wild-type and *hog1*Δ cells had similar total levels of mannan and chitin, cells lacking Hog1 had more exposed chitin (Fig. 3F), which may underlie the increased resistance to calcofluor white. Altogether, these results indicate that Hog1 plays key roles in cellular morphology, aggregation, and cell wall structure in C. auris.

### Hog1-mediated stress resistance in C. auris.

**FIG 4:**
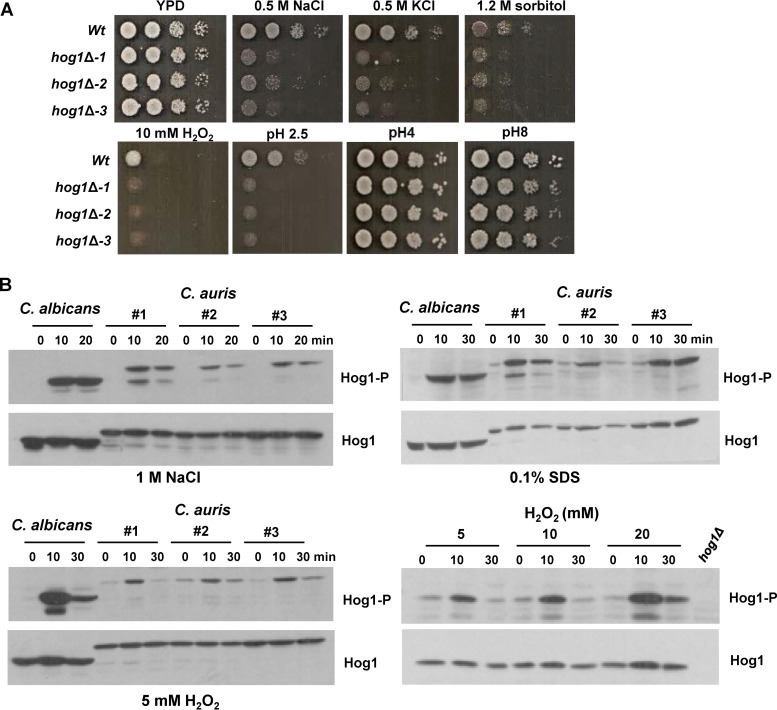
Stress-protective roles of *C. auris* Hog1. (A) Hog1 is required for resistance to diverse stresses. Exponentially growing cells were spotted in serial dilutions onto YPD agar plates containing the indicated additives and incubated for 24 or 48 h at 30°C. (B) Hog1 is activated in response to diverse stresses. Western blots depicting Hog1 phosphorylation in response to the indicated stresses. Blots were probed for phosphorylated Hog1 (Hog1-P), stripped, and reprobed for total Hog1 (Hog1).

To determine stress-protective roles of the Hog1 SAPK in C. auris, the relative tolerance of *hog1*Δ cells to diverse stresses was examined. Cells lacking Hog1 were sensitive to cationic stress imposed by either NaCl or KCl and to osmotic stress imposed by sorbitol (Fig. 4A). Hog1 was also required for the resistance to the reactive oxygen species H_2_O_2_ and to highly acidic environments (Fig. 4A). However, Hog1 was dispensable for growth in alkaline and moderately acidic environments (Fig. 4A). C. auris Hog1 was also dispensable for the resistance to the organic oxidative stress-inducing agent *tert*-butyl hydroperoxide, the organic acid sorbic acid, fluconazole, and nitrosative stress induced by sodium nitrite (see Fig. S3). To explore whether Hog1 was activated in response to the same panel of stresses that require this SAPK for resistance, Western blotting of cell extracts was performed using an antibody that recognizes the active (phosphorylated) form of Hog1. Samples from C. albicans were included as controls. As shown in Fig. 4B, Hog1 in all three C. auris wild-type isolates was phosphorylated in response to cationic stress, oxidative stress, and SDS stress. The level of Hog1 phosphorylation in C. auris following cationic or oxidative stress was less than that observed in C. albicans. This could be due to less Hog1 protein in C. auris or to the fact that this pathogen is more resistant than C. albicans to both cationic and oxidative stresses (Fig. 1A). Indeed, the exposure of C. auris to increasing H_2_O_2_ concentrations resulted in higher levels of Hog1 phosphorylation (Fig. 4B). Altogether, these data indicate that the activation of C. auris Hog1 in response to osmotic, oxidative, and SDS-imposed stresses is physiologically important, as cells lacking Hog1 are much less tolerant of such stresses.

10.1128/mSphere.00506-18.3 FIG S3 Hog1-independent stress resistance. Exponentially growing cells were spotted in serial dilutions onto YPD agar plates containing the indicated additives and incubated for 24 or 48 h at 30°C. Download FIG S3, TIF file, 2.8 MB. Copyright © 2018 Day et al. 2018 Day et al. This content is distributed under the terms of the Creative Commons Attribution 4.0 International license.

### Hog1 is required for virulence in C. auris.

**FIG 5:**
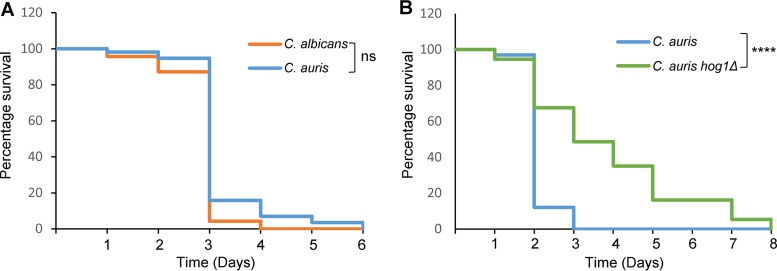
*C. elegans* model of infection. (A) C. auris displays comparable virulence to C. albicans in C. elegans. (B) Deletion of Hog1 attenuates C. auris virulence in C. elegans. In both experiments, nematodes were infected with the indicated strains and the survival was monitored daily. These data are from a single experiment; two further independent biological replicates are shown in Fig. S4 in the supplemental material.

To investigate the role of Hog1 in C. auris virulence, we employed the invertebrate model host Caenorhabditis elegans (36). This model has been used successfully to investigate C. albicans virulence, as feeding the nematodes live, but not heat killed, fungal cells rapidly kills the host (37) and strains showing attenuated virulence in murine models of systemic infection similarly show diminished virulence in the C. elegans model (37, 38). Initially, we determined whether C. auris was pathogenic toward C. elegans and compared its virulence to that of C. albicans. Similar to that reported in Galleria mellonella (11) and in systemic mouse models of infection (12), C. auris killed the C. elegans host as effectively as C. albicans (Fig. 5A and S4). Next, we compared the virulence of C. auris wild-type and *hog1*Δ cells. The survival of C. elegans was significantly extended after infection with *hog1*Δ cells compared to that with wild-type cells (*P < *0.001) (Fig. 5B and S4). Taken together, these results illustrate that the invertebrate model host can be used to study C. auris virulence and that the Hog1 SAPK is an important pathogenicity determinant in this emerging fungal pathogen of humans.

10.1128/mSphere.00506-18.4 FIG S4 C. elegans model of infection. Two independent biological replicates showing that C. auris displays comparable virulence to C. albicans, and that deletion of Hog1 attenuates C. auris virulence in C. elegans. In both experiments nematodes were infected with the indicated strains and survival monitored daily. Download FIG S4, TIF file, 1.4 MB. Copyright © 2018 Day et al. 2018 Day et al. This content is distributed under the terms of the Creative Commons Attribution 4.0 International license.

## DISCUSSION

A phenotypic analysis of C. auris revealed this fungal pathogen to have a distinct stress resistance profile. Compared to the other pathogenic *Candida* species examined, C. auris displayed the greatest tolerance to cationic stress and yet the least resistance to the superoxide-generating drug menadione (vitamin K) and the organic peroxide *tert-*butyl hydroperoxide (*t*-BOOH). It is not clear whether such sensitivity is due to the specific ROS liberated by menadione and *t*-BOOH or to the fact that both are organic compounds. Moreover, although relatively resistant to H_2_O_2_ as shown previously (21), C. auris was acutely sensitive to H_2_O_2_ in combination with cationic stress. Such combinatorial stress-mediated synergistic killing has been documented in a range of model yeasts and fungal species and attributed to the prevention of the induction of oxidative stress-protective genes (26, 27). The identification of single, and combinations of, environmental stresses that C. auris is acutely sensitive to may inform the development of more efficient disinfection strategies to eradicate the persistence of this fungus within hospital settings. It is also notable that C. auris fails to grow under anaerobic conditions and exhibits impaired growth in acidic environments. These observations argue against C. auris being a resident commensal organism within the human gut. Consistent with this, while C. auris has been detected at multiple body sites such as skin, nose, axilla, groin, and rectum (3), there are no reports of gut colonization.

After the characterization of stress resistance phenotypes in C. auris, we sought to investigate the role of the Hog1 SAPK in stress sensing and signaling by generating strains lacking the *HOG1* gene. The first successful gene knockout in C. auris was reported recently, in which an expression-free CRISPR-Cas9 system was used to create strains lacking the catalase gene *CTA1* (10). In this study, the inclusion of purified CRISPR RNA-Cas9 protein complexes (RNPs) was found to significantly enhance the efficiency of generating gene knockouts in C. auris and other haploid *Candida* species. Specifically, the use of RNPs increased the number of accurate transformants in which the C. auris *CTA1* gene was deleted from 50% to 70% (10). Here, we employed the same nourseothricin resistance marker selection, although only 100 bp of flanking sequence homology was incorporated into the disruption cassette instead of the 1 kb employed in the previous study (10). Nonetheless, 30% of the nourseothricin-resistant (NAT^r^) transformants screened were accurate haploid transformants in which the *HOG1* gene was deleted. Thus, gene deletions can be made in C. auris using standard techniques (35) without the CRISPR system or the incorporation of large flanking regions of sequence homology into the disruption cassette.

C. auris Hog1 is activated and promotes stress resistance to diverse stimuli, including osmotic stress, the oxidizing agent H_2_O_2_, and the denaturing agent SDS. The role of fungal SAPK pathways in promoting osmotic stress tolerance is universal (17), and a role in oxidative stress protection has been identified in a range of model (39, 40) and pathogenic (30, 31, 41–43) fungi. Similarly, Hog1 signaling that confers protection to the denaturant SDS has also been reported for a number of pathogenic fungi (30, 42, 44). In contrast, although required for growth in highly acidic environments, no requirement for C. auris Hog1 in weak acid tolerance was detected. This is in contrast to that reported in C. glabrata, in which a recent paper revealed that Hog1-mediated tolerance to lactic acid enables cocolonization with *Lactobacillus* spp. (45). Furthermore, no role for C. auris Hog1 was found for resistance to nitrosative stress, unlike that recently reported for C. albicans Hog1 (46). Such differences in the activation profile of C. auris Hog1 compared to those in other human-pathogenic fungi may reflect the different environmental niche of this emerging pathogen.

In addition to the impaired stress resistance phenotypes, we amassed data indicative of Hog1 regulating the C. auris cell wall. Cells lacking *HOG1* had more exposed chitin than wild-type cells and displayed greater resistance to the chitin-binding dye calcofluor white. In addition, *hog1*Δ cells were more resistant to the β-1,3-glucan binding dye Congo red and to the echinocandin antifungal caspofungin, which targets β-1,3-glucan synthase. An increased resistance to cell wall inhibitors is also seen in C. albicans *hog1*Δ cells, and this has been attributed to cross talk between mitogen-activated protein kinase (MAPK) pathways, resulting in the inappropriate activation of the Cek1 cell wall integrity MAPK (47). Given the presence of an orthologue of Cek1 in C. auris (79.9% identical to C. albicans Cek1), it is tempting to speculate that a similar mechanism underlies the cell wall changes in C. auris *hog1*Δ cells. Notably, Hog1-modulated cell wall remodeling in C. albicans impacts host-pathogen interactions. For example, cell wall remodeling triggered by neutrophil extracellular traps requires a functional Hog1 pathway (48), and more recently, it was shown that Hog1 regulation of cell wall remodeling is required for the initiation of pyroptosis following macrophage engulfment (49).

SAPKs have been implicated in a range of cellular processes in pathogenic fungi in addition to promoting stress resistance. For example, the Hog1 SAPK in C. albicans functions to regulate cytokinesis in budding cells (18), white opaque switching (50), and morphogenetic switching between yeast and filamentous forms (51). In Cryptococcus neoformans, Hog1 modulates morphological differentiation during mating and the production of two key virulence determinants, melanin and capsule (31). In C. glabrata, Hog1 has been shown to regulate adhesion of this pathogenic yeast in a mechanism involving iron homeostasis (30). Following the same trend, we found that C. auris Hog1 regulates traits in addition to stress resistance. Notably, C. auris cells lacking *HOG1* were larger and more elongated than wild-type cells, suggesting that a functional SAPK pathway is required for normal cell cycle progression. In addition, *hog1*Δ cells were highly flocculent and formed large aggregates. This may be related to the cell wall alterations in *hog1*Δ cells, but the precise mechanism underlying this requires further investigation. In addition, it is not clear whether the mechanism underlying aggregation in *hog1*Δ cells is related to that underlying the aggregation phenotype previously documented in a subset of clinical isolates (11). However, it is interesting, in view of the attenuated virulence exhibited by *hog1*Δ cells, that non-aggregate-forming isolates of C. auris were found to be more virulent than aggregate-forming isolates (11).

Finally, we present evidence that the Hog1 SAPK is an important virulence trait of C. auris in the replacement, reduction, and refinement (3R)-compliant (52) C. elegans infection model. To the best of our knowledge, this is the first report of a factor required for the virulence of this emerging pathogen. Notably, Hog1 homologues are emerging as a universal virulence factor in pathogenic fungi, as *hog1*Δ mutants display attenuated virulence in all major human fungal pathogens (18, 29–31). This underscores the importance of fungal stress responses in promoting pathogenesis and suggests that the development of drugs which target fungal SAPK pathways has the exciting potential to generate broad-acting antifungal treatments for human mycoses.

## MATERIALS AND METHODS

### Strains and growth conditions.

Three clinical C. auris isolates were provided by the U.K. National Mycology Reference Library, Public Health England; NCPF8985, NCPF8971, and NCPF8977 (53). Clinical reference strains of C. albicans (SC5314 [54]), C. dubliniensis (CD36 [55]), and C. glabrata (CBS-128 [56]) were used for comparison. The strains were grown in YPD medium (2% yeast extract, 1% Bacto peptone, 2% glucose) at 30°C.

The C. auris *hog1*Δ mutant was constructed using the Clox system with nourseothricin selection, developed for use in C. albicans (35). However, due to the haploid nature of C. auris (9), only one round of transformation was necessary. The *NAT1-*Clox disruption cassette was PCR amplified using Extensor master mix (Thermo Scientific, MA, USA) with the chimeric primers CaurisHog1delF2 (TTTTACCCTTTCTACCCTTCGCTATACCGCCTTCGGGAGGAATCTCGCAACAAACCACAGCCAGCCAAAATAAGCCACTAACTGCTTCGTTACTTCCTCGACGGCCAGTGAATTGTAATA) and CaurisHog1delR2 (TTGATGTTTTAAAAGTCATGAGCGAAACTGACACATGTGTCTGTCA CTACCCAATGTCTATCTGACTCAAGTATCATAAAATCAAACTCCTGCAAACGCATCGGAATTAACCCTCACTAA). The sequences are homologous to the 5′ and 3′ flanking regions of the C. auris *HOG1* gene, with the underlined sequences homologous to Clox landing pad sequences (35). The resulting disruption cassette was then used to transform C. auris NCPF8985. The transformation method used was modified from that of Schiestl and Gietz (57). Briefly, 5 × 10^8^ cells were harvested and washed in 20 ml LiAcTE buffer (0.1 M lithium acetate [LiAc; pH 7.5], 0.1 M Tris-HCl [pH 7.5], 0.01 M EDTA). Cells were resuspended in 1 ml LiAcTE, and 100 µl was aliquoted for each transformation. One hundred micrograms of salmon sperm carrier DNA and 5 µg of the DNA disruption cassette were added, followed by 700 µl 40% polyethylene glycol 3350 (PEG 3350) in LiAcTE, and the cells were incubated with agitation at 30°C for 3 h. The cells were heat shocked by incubation at 42°C for 45 min, harvested by centrifugation, and plated on nourseothricin-containing YPD medium (200 µg/ml) supplemented with 2.5 mM methionine and 2.5 mM cysteine to repress *MET3*_p_*-cre* expression. The marker was then resolved by growing transformants on Sabouraud dextrose (SD) medium without methionine or cysteine supplementation. The successful integration of the resolved disruption cassette was detected by diagnostic PCR using the primers CaurisHogdelChF2 (ATTTGAGACACCTCCAGCTTCGCC) and loxPR (TTCGTATAATGTATGCTATACG). Following a single round of transformation, three independent C. auris *hog1*Δ strains (JC2310, JC2311, and JC2312) were successfully created using this approach.

### Stress resistance assays.

*Candida* strains were grown at 30°C to mid-exponential phase, and then 10-fold serial dilutions were spotted using a 48-prong replica plater (Sigma-Aldrich) onto YPD plates containing the indicated compounds. The plates were incubated at 30°C for 24 to 48 h. To quantify survival in liquid cultures, 25 mM H_2_O_2_ was added to exponentially growing cells. The cells were taken at various time points, diluted, and then plated on YPD agar to determine the numbers of surviving cells. The plates were incubated at 30°C for 24 h to 48 h, and survival was expressed as a percentage of the time zero sample. The experiments were repeated three times.

### Western blotting.

Exponentially growing cells (25 ml) were harvested by centrifugation (3,000 rpm for 1 min), before and after exposure to the indicated stress agent. The supernatants were discarded, and the pellets were snap-frozen in liquid nitrogen. The pellets were thawed and washed in ice-cold lysis buffer (20 mM HEPES [pH 7.3], 350 mM NaCl, 10% glycerol, 0.1% Tween 20) containing protease inhibitors (2 μg/ml pepstatin A, 2 μg/ml leupeptin, 1 mM phenylmethylsulfonyl fluoride [PMSF], 20 μg/ml aprotinin) and phosphatase inhibitors (2 mM sodium orthovanadate, 50 mM sodium fluoride). The cells were resuspended in 200 μl lysis buffer and transferred to a ribolyser tube containing 1 ml chilled glass beads. The samples were disrupted by bead beating (BioSpec) for 2 × 15 s. Lysates were recovered by centrifugation, and 30 µg of extract was subjected to SDS-PAGE on 10% gels. Hog1 was detected by Western blot analysis using an anti-Hog1 antibody (y-215; Santa Cruz Biotechnology). Phosphorylated Hog1 was detected with an anti-phospho-p38 antibody (9211; Cell Signaling Technology) as described previously (43). Protein loading in some experiments was determined using an anti-tubulin antibody (DSHB, University of Iowa). The experiments were performed three times.

### Microscopy.

To image wild-type and C. auris *hog1*Δ cells, exponentially growing cells were fixed in 3.7% (wt/vol) paraformaldehyde and spread on poly-l-lysine-coated slides. The cells were mounted onto slides using Vectashield mounting medium containing 1.5 mg/ml 4′-6-diamidino-2-phenylindole (DAPI) (Vector Laboratories, Burlingame, CA). Differential interference contrast (DIC) images were captured using a Zeiss Axioscope as described previously (43). For cell wall staining experiments, C. auris cells were fixed with 10% (vol/vol) neutral buffered formalin solution (Sigma-Aldrich). Next, 2 × 10^6^ fixed cells were exposed to 100 µg/ml rhodamine concanavalin A (Vector Laboratories, Burlingame, CA) for 45 min to stain mannan, 100 µg/ml fluorescein isothiocyanate (FITC)-conjugated wheat germ agglutinin (WGA; Sigma-Aldrich) for 60 min to stain exposed chitin, and to 25 µg/ml calcofluor white (CFW; Sigma-Aldrich) for 3 min to stain cell wall total chitin (58). Vectashield mounting medium was added to each sample to preserve the fluorescence. The samples were visualized by DIC field and by fluorescence imaging using a standard FITC and DAPI filter set (Chroma Technology Corporation) on a DeltaVision Core microscope (Applied Precision). Images were taken using a QuantEM:512SC camera and analyzed with DeltaVision software (SoftWorx version 5.0.0). The exposure time used when capturing fluorescence images was kept constant across all samples to enable relative chitin content to be compared.

### C. elegans pathogenesis assay.

Wild-type C. elegans (N2) was used throughout the experiment and was maintained on nematode growth medium (NGML) with E. coli OP50 as the food source as described previously (36). Worms were synchronized via egg lay (59) and allowed to develop to the L4 stage by incubating at 25°C for 2 days. Approximately 50 worms were transferred to unseeded NGML plates and incubated at 25°C for 1 h to minimize the transference of E. coli before being transferred to brain heart infusion (BHI) plates seeded with either C. albicans wild-type (SC5314), C. auris wild-type (NCPF8985), or C. auris *hog1*Δ (JC2310) cells and containing 150 μM 5-fluoro-2′-deoxyuridine (FUdR) (Sigma) to inhibit reproduction (60). The plates were incubated at 25°C. The worms were examined daily, and worms that showed no pharyngeal contraction and did not move in response to probing with a pick were scored as dead and removed from the plate. Differences in C. elegans survival were determined by the log rank test. In all experiments, a *P* value of <0.05 was considered significant.
