# Supplementary figures and images for: Hog1 Regulates Stress Tolerance and Virulence in the Emerging Fungal Pathogen Candida auris
Source: mSphere. 2018 Oct 24;3(5):e00506-18. doi: 10.1128/mSphere.00506-18 (PMC6200985; doi:10.1128/mSphere.00506-18)

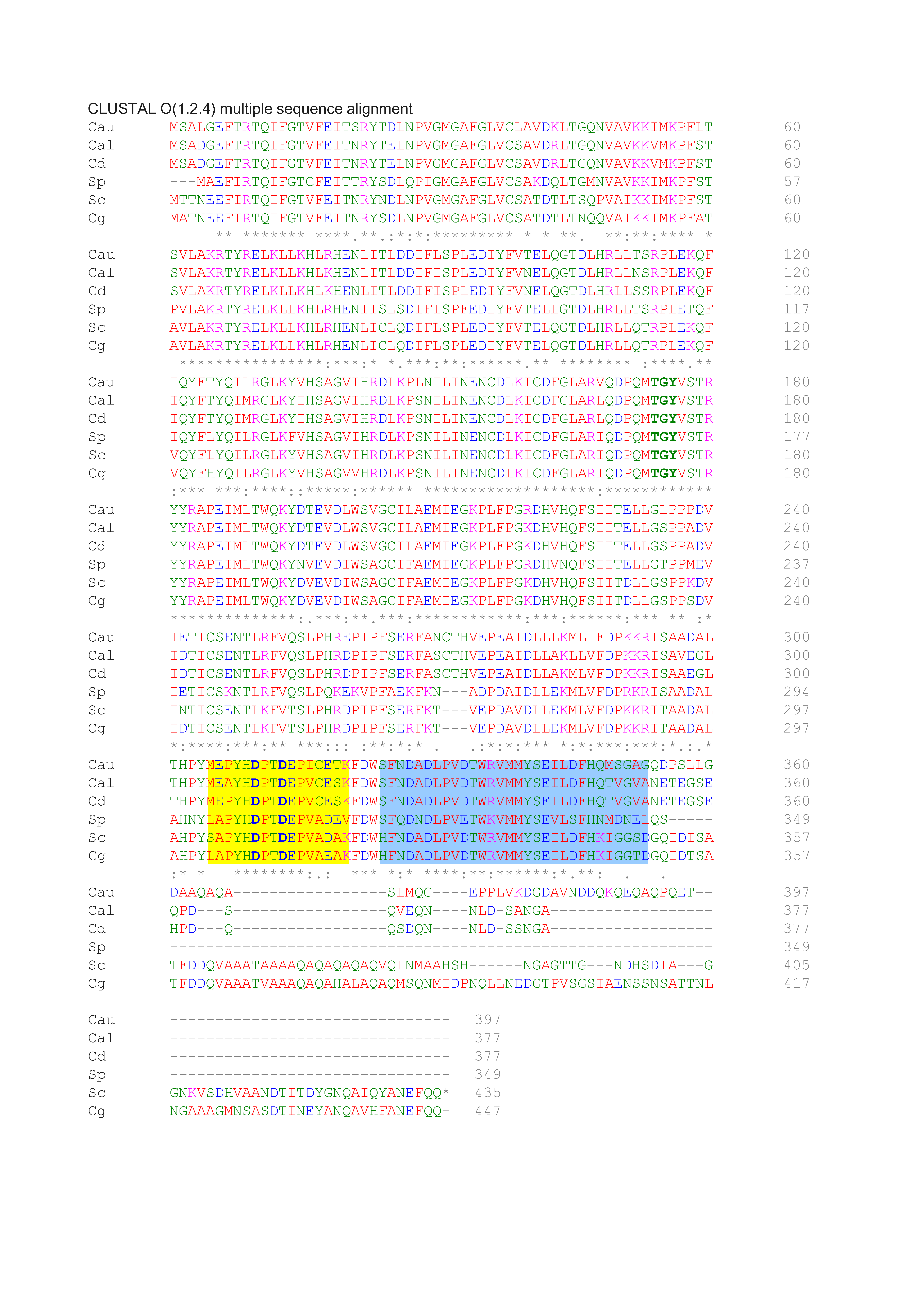

Supplement: FIG S1 [file sph005182664sf1.tif]

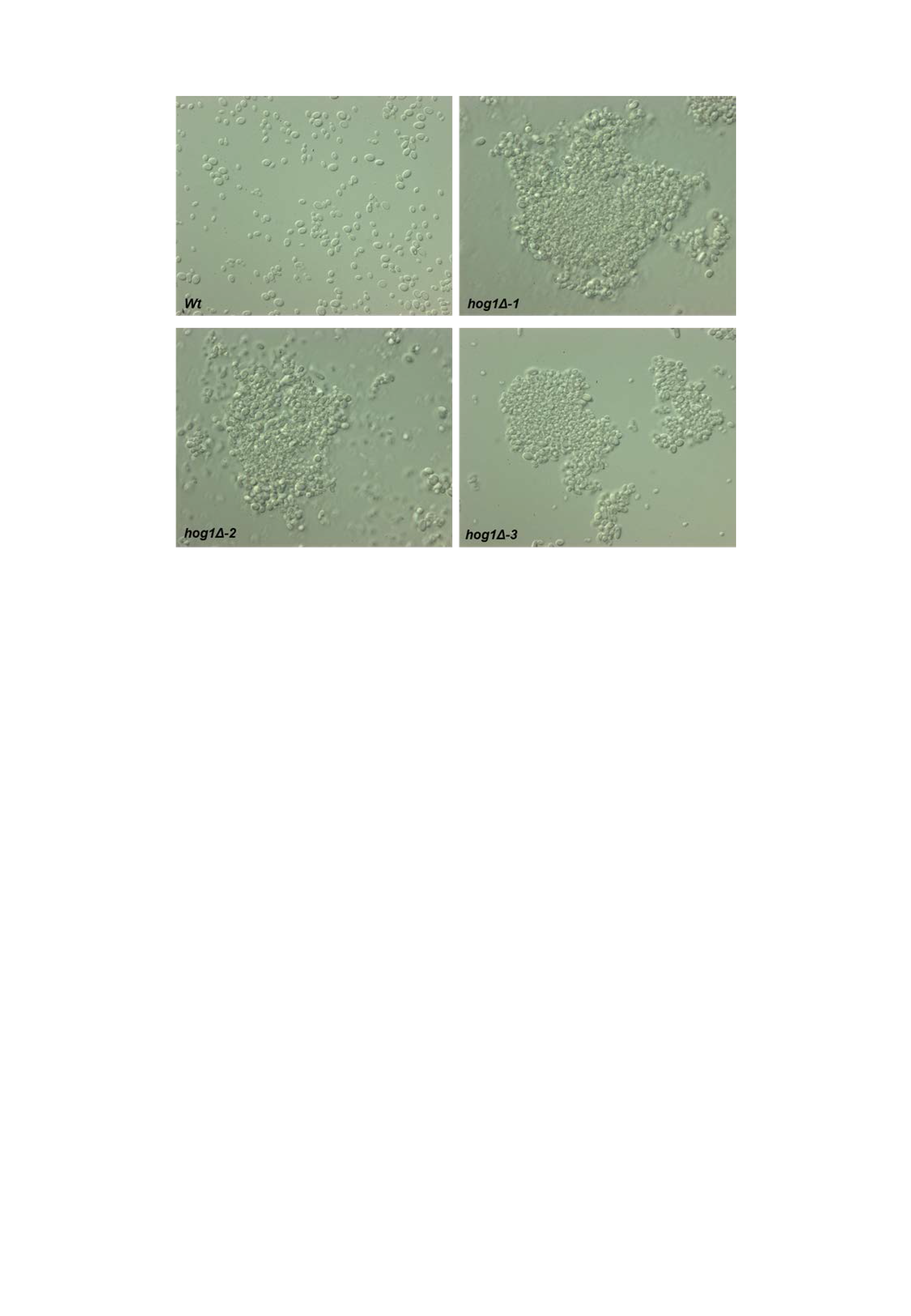

Supplement: FIG S2 [file sph005182664sf2.tif]

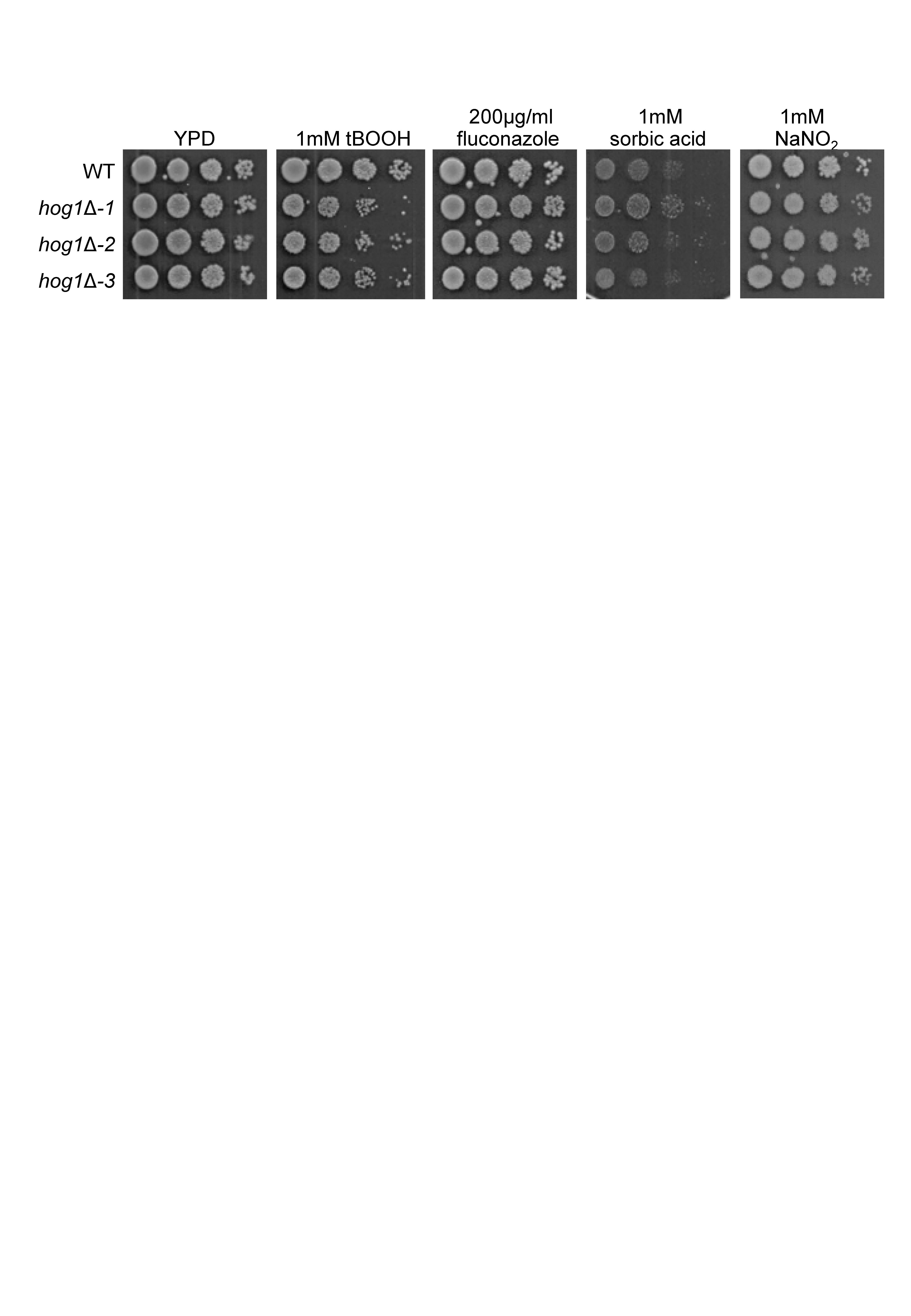

Supplement: FIG S3 [file sph005182664sf3.tif]

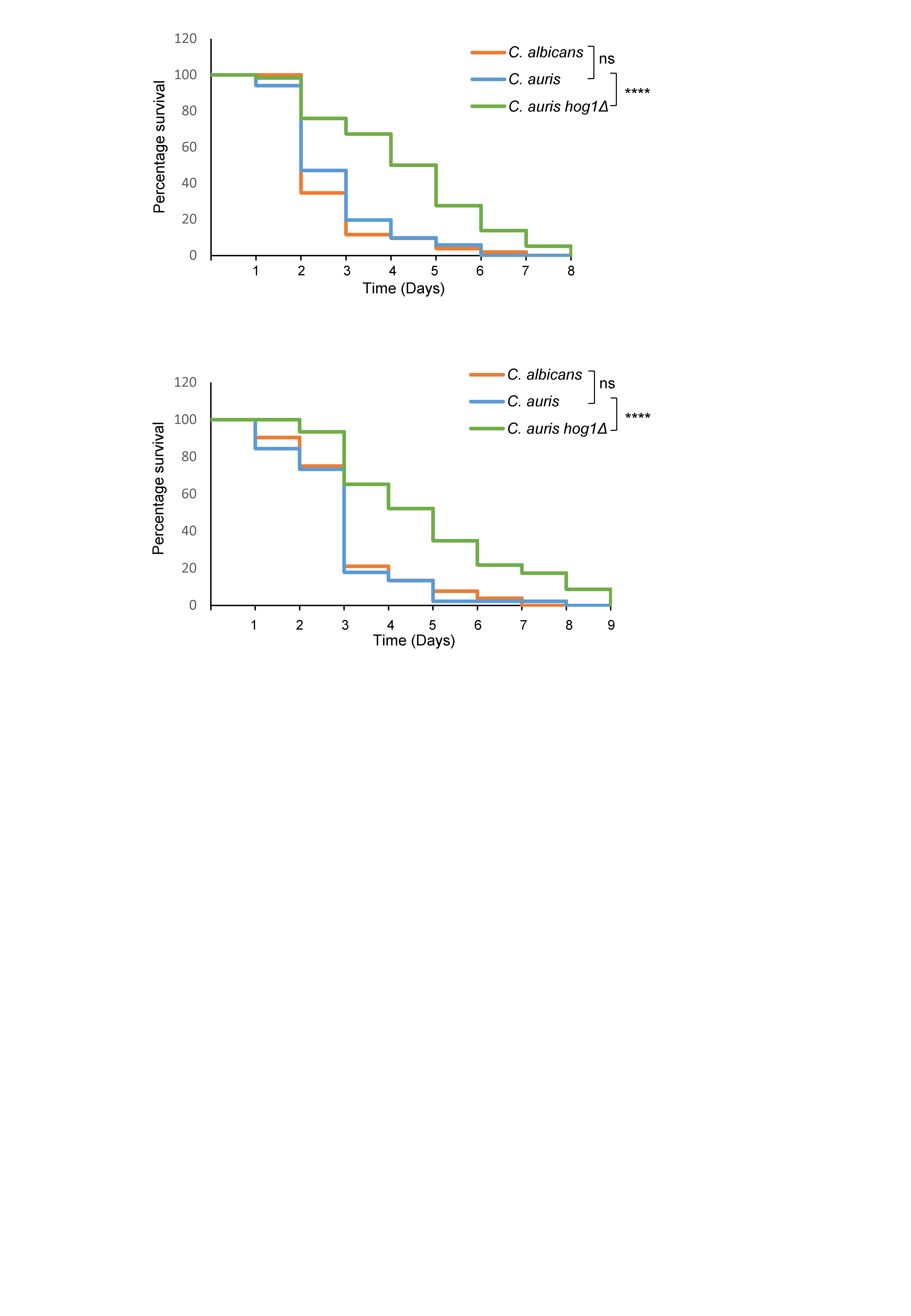

Supplement: FIG S4 [file sph005182664sf4.tif]
